# Purinergic Signaling in Pulmonary Inflammation

**DOI:** 10.3389/fimmu.2019.01633

**Published:** 2019-07-16

**Authors:** Thanh-Thuy T. Le, Nathaniel K. Berg, Matthew T. Harting, Xiangyun Li, Holger K. Eltzschig, Xiaoyi Yuan

**Affiliations:** ^1^Department of Anesthesiology, McGovern Medical School, University of Texas Health Science Center at Houston, Houston, TX, United States; ^2^Department of Pediatric Surgery, McGovern Medical School, Children's Memorial Hermann Hospital, The University of Texas Health Science Center at Houston, Houston, TX, United States; ^3^Department of Anesthesiology, Tianjin Nankai Hospital, Tianjin Medical University, Tianjin, China

**Keywords:** purinergic signaling, nucleotides, ectonucleotidase, adenosine, acute pulmonary inflammation, chronic pulmonary inflammation

## Abstract

Purine nucleotides and nucleosides are at the center of biologic reactions. In particular, adenosine triphosphate (ATP) is the fundamental energy currency of cellular activity and adenosine has been demonstrated to play essential roles in human physiology and pathophysiology. In this review, we examine the role of purinergic signaling in acute and chronic pulmonary inflammation, with emphasis on ATP and adenosine. ATP is released into extracellular space in response to cellular injury and necrosis. It is then metabolized to adenosine monophosphate (AMP) via ectonucleoside triphosphate diphosphohydrolase-1 (CD39) and further hydrolyzed to adenosine via ecto-5′-nucleotidase (CD73). Adenosine signals via one of four adenosine receptors to exert pro- or anti-inflammatory effects. Adenosine signaling is terminated by intracellular transport by concentrative or equilibrative nucleoside transporters (CNTs and ENTs), deamination to inosine by adenosine deaminase (ADA), or phosphorylation back into AMP via adenosine kinase (AK). Pulmonary inflammatory and hypoxic conditions lead to increased extracellular ATP, adenosine diphosphate (ADP) and adenosine levels, which translates to increased adenosine signaling. Adenosine signaling is central to the pulmonary injury response, leading to various effects on inflammation, repair and remodeling processes that are either tissue-protective or tissue destructive. In the acute setting, particularly through activation of adenosine 2A and 2B receptors, adenosine signaling serves an anti-inflammatory, tissue-protective role. However, excessive adenosine signaling in the chronic setting promotes pro-inflammatory, tissue destructive effects in chronic pulmonary inflammation.

## Introduction

As one of the most ancient signaling pathways, purinergic signaling is driven by the heterocyclic aromatic compounds known as purines. The purine nucleotide adenosine triphosphate (ATP) functions as the energy currency at the very foundation of mammalian biologic reactions. In this review, we examine the roles of purine nucleotides and nucleosides and purinergic signaling in acute and chronic pulmonary inflammation, with emphasis on ATP, adenosine diphosphate (ADP), and adenosine (1). In 1929, Drury and Szent-Gyorgyi first reported evidence of purinergic signaling when they observed an adenine compound from cardiac extracts caused transient heart block when injected intravenously into live animals (2). Today, adenosine is still utilized clinically for its ability to slow the heart rate. Research over the last few decades has elucidated various biologic effects of ATP, ADP, and adenosine. We will focus on their effects in pulmonary injury and inflammation, in disease settings such as acute and chronic pulmonary inflammation.

### Acute Respiratory Distress Syndrome

Acute respiratory distress syndrome (ARDS), previously known as acute lung injury (ALI), is a life-threatening condition that is a common cause of respiratory failure, morbidity, and mortality in critically ill patients (3, 4). ARDS can occur secondary to a number of insults, including pneumonia, aspiration, trauma, and sepsis (5). Less common causes of ARDS include acute pancreatitis, administration of blood products (transfusion-associated acute lung injury or TRALI), drug overdose, near drowning, reperfusion injury, hemorrhagic shock, and post-lung transplant graft dysfunction (6). ARDS is clinically defined as acute onset (within 1 week of insult) of respiratory failure, noncardiogenic pulmonary edema (bilateral opacities on chest imaging), and hypoxemia (as defined by PaO2/FiO2 < 300 mmHg with a minimum positive end-expiratory pressure (PEEP) of 5 cm H_2_O) (7). The pathophysiology of ARDS is characterized by acute pulmonary inflammation including diffuse alveolar damage (with both alveolar epithelial and endothelial injury present), increased pulmonary vascular permeability resulting in accumulation of protein-rich interstitial and bronchoalveolar space edema, and excessive recruitment and infiltration of immune cells (6, 8). There are no specific therapies for ARDS currently and mortality is 30–40% (6). In this review, we will discuss the role of purinergic signaling in acute pulmonary inflammation seen in ARDS.

### Chronic Lung Diseases

Chronic lung diseases, such as asthma, chronic obstructive pulmonary disease (COPD), and idiopathic pulmonary fibrosis (IPF), develop as a result of non-resolvable pulmonary inflammation and dysregulated tissue remodeling that lead to a progressive decline in respiratory function (9–11). Chronic lung diseases are prevalent and lethal, ranking just behind cancer and cardiovascular disease in mortality rate in the United States. Therapies for symptomatic control in these diseases are available but none is able to reverse or cure the aberrant wound healing and remodeling seen in the lungs of these patients. Though the causes of chronic lung diseases are varied, a common feature among these conditions is excessive recruitment and dysregulated activation of effector cells, including neutrophils, eosinophils, macrophages, airway epithelial cells (AECs), fibroblasts and myofibroblasts, leading to release of more mediators that potentiate pulmonary inflammation and remodeling (9–11). Prominent features seen in chronic lung diseases include excessive angiogenesis, airway epithelial cell remodeling and deposition and metabolism of extracellular matrix (10, 12). In asthma, there is excessive bronchial collagen deposition and angiogenesis and thickened basement membrane. In IPF, fibroblast proliferates excessively and deposits copious extracellular matrix in alveolar airways. In COPD, alveolar airways are destroyed by an imbalance between proteases and anti-proteases governing matrix breakdown. Inflammation, angiogenesis, and matrix deposition and breakdown are a part of the normal wound healing process. However, in chronic lung diseases, these processes are overactive or dysregulated, leading to disease (10, 11).

### The Role of Purinergic Signaling in Acute and Chronic Pulmonary Inflammation

Inflammatory and hypoxic conditions lead to increased release of ATP/ADP, which translates to elevated extracellular adenosine levels (1, 13). Hypoxia and hypoxia-inducible factors (HIFs) further support the increase in extracellular adenosine via transcriptional regulation of adenosine metabolizing and receptor genes (14–19). The significance of extracellular adenosine metabolism is evident in mice with genetic deletion of ectonucleoside triphosphate diphosphohydrolase-1 (CD39) and ecto-5'-nucleotidase (CD73) resulting in decreased adenosine concentration and signaling despite elevated or normal ATP levels. These mice have been shown to exhibit enhanced mucosal inflammation (20, 21). The exaggerated inflammation is prevented, however, in the presence of ENT or AK inhibitors (inhibiting termination of adenosine signaling) (22–24). Adenosine signaling is central to injury response in the lung. Via engagement of cell surface G-protein-coupled adenosine receptors, adenosine exerts various effects on inflammation, repair, and remodeling processes (25), producing either tissue-protective or tissue destructive results (11, 26). Adenosine serves an anti-inflammatory, tissue-protective role [particularly through activation of the A2A and A2B adenosine receptors (A2AAR, A2BAR)] in acute lung injury (26, 27). However, chronically elevated adenosine, with activation of A1 adenosine receptor (A1AR), A2BAR, and A3 adenosine receptor (A3AR), promotes a pro-inflammatory state and excessive, dysregulated tissue remodeling that contributes to development and progression of chronic lung diseases (11, 26).

## Biology of Purinergic Signaling

### Extracellular Nucleotide Release and Signaling

**Figure 1:**
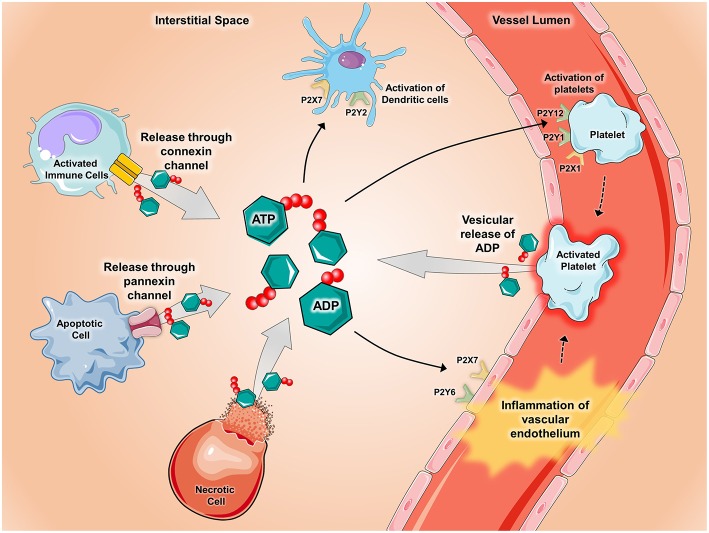
Release of ATP during inflammatory conditions. During conditions of inflammation such as during ischemia-reperfusion injury, hypoxia, inflammatory bowel disease, acute lung injury, and vascular thrombosis, ATP and ADP are released into the extracellular space via several mechanisms. ATP and ADP are released from apoptotic cells through pannexin-hemichannels and from connexin-hemichannels located on activated immune cells. Additionally, ATP and ADP can be released after cell lysis occurs in necrotic cells and though vesicular release by activated platelets. Once released, ATP and ADP act as potent signaling molecules by binding to and activating P2X receptors (ligand-gated ion channels) and P2Y receptors (G-protein-coupled receptors). Pictured are several examples of how ATP and ADP activate P2x and P2Y receptors during inflammatory states. P2Y6 and P2X7 receptors on vascular endothelium promote inflammation whereas activation of the P2X1, P2Y1, and P2Y12 receptors mediate platelet activation. In the setting of chronic lung diseases such as asthma, P2X7 and P2Y2 receptors promote activation of dendritic cells. Components of the figure were modified from SMART Servier Medical Art Library.

The roles of purine nucleotides, nucleosides and purinergic signaling in acute and chronic pulmonary inflammation have been studied extensively, with emphasis on ATP, ADP, and adenosine. ATP can be found at physiologic concentrations in mammalian cells at baseline and the release/accumulation of ATP in disease states are summarized in Figure 1. In disease states, such as in inflammation and ischemia, ATP is released from intracellular stores due to cellular necrosis (28). During apoptosis, pannexin hemichannels control ATP release into the extracellular space, where ATP serves as a phagocyte chemotactic signal (29). Inflammatory cells (like neutrophils) and endothelial cells can release ATP via connexin hemichannels (30–32). ADP can be secreted from intracellular granules by platelets (28). ATP signals through receptors initially designated P2 receptors (33), then later re-classified into P2X receptors (ligand-gated ion channels) and P2Y receptors (G-protein-coupled receptors). Mice with genetic deletion of P2 receptors are viable and protected from inflammatory diseases such as asthma, vascular inflammation and graft-vs-host disease (34–36). Pharmacologic antagonism of P2 receptors resulted in inhibition of inflammation in inflammatory bowel diseases (IBD), pulmonary inflammation, and ischemia-reperfusion injury (28, 35, 37).

### Extracellular Conversion of ATP and ADP to Adenosine

**Figure 2:**
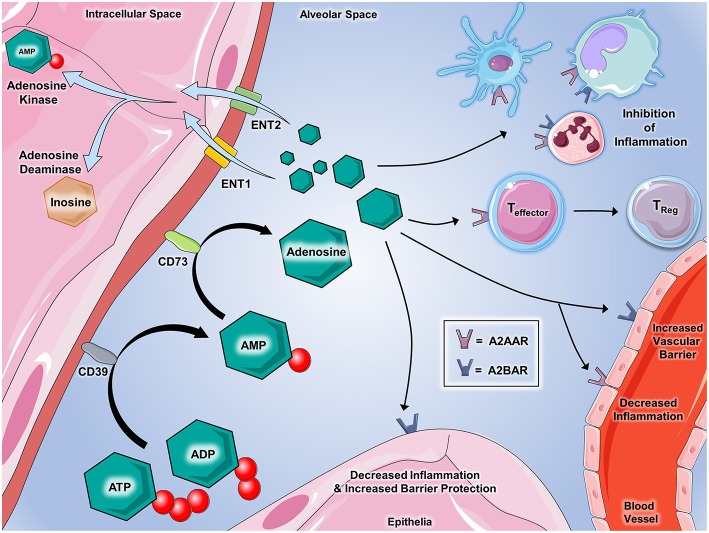
Extracellular adenosine signaling and its termination. Adenosine partakes in a number of signaling events during inflammatory conditions. ATP and ADP that is released serve as the source of extracellular adenosine. CD39 (Ecto-nucleotide triphosphate diphosphohydrolase 1, E-NTPDase1) dephosphorylates extracellular ATP and ADP on the cell surface to generate AMP, which is further dephosphorylated by CD73 (ecto-5'-nucleotidase, Ecto5'NTase) into adenosine. Once generated by enzymatic dephosphorylation, adenosine plays several important roles in regulating inflammation and immunity. Binding with A2AAR and A2BAR on immune cells promotes the inhibition of inflammation mediated by a number of innate immune cells including dendritic cells, monocytes, macrophages, and neutrophils. Adenosine interaction with A2AAR on T cells has demonstrated the suppression of effector functions and promotes the transition to T-regulatory status. On vascular endothelial cells, A2AAR and A2BAR activation decreases cellular inflammatory responses and promotes the integrity of barrier functions, respectively. Epithelial cells from several tissues including lung, gastrointestinal, myocardial, and renal contain A2BAR that, when activated by adenosine, are shown to play critical roles in decreasing inflammation and promoting barrier integrity during inflammation and injury. Several mechanisms are involved in regulating adenosine in order to allow for appropriate termination of signaling. Equilibrative nucleoside transporters (ENT)-1 and−2 deplete the extracellular accumulation of adenosine by transporting it into the nucleus. Adenosine kinase and adenosine deaminase are enzymes that both act to “inactivate” adenosine and inhibit its ability to bind to its receptors. Adenosine deaminase converts adenosine back to AMP and adenosine deaminase converts adenosine to inosine, which is an important step in the metabolism of nucleotides. Components of the figure were modified from SMART Servier Medical Art Library.

Extracellular ATP and ADP are swiftly converted to adenosine monophosphate (AMP), which is then further metabolized to adenosine (Figure 2). This two-step nucleotide phosphohydrolysis is mediated by ectoenzymes. Ecto-nucleoside triphosphate disphosphohydrolases (E-NTPDases), which include CD39, regulate the conversion of ATP and ADP to AMP. Metabolism of AMP to adenosine occurs via CD73. CD39 deficient (*CD39*^−/−^) mice are viable and exhibit increased ATP and ADP levels along with decreased adenosine levels leading to increased risk of developing uncontrolled, disordered inflammation (38–40). Decreased expression of CD39 can be seen in humans with polymorphisms of CD39 noncoding regions, which confers increased susceptibility to the development of irritable bowel disease and multiple sclerosis (41, 42). Loss-of-function mutations in the CD73 gene in human result in familial peripheral artery calcifications (43). Mice deficient in CD73 (*CD73*^−/−^) are viable, and during disease states, exhibit lower levels of adenosine signaling despite ATP and ADP levels being nearly unchanged. *CD73*^−/−^ mice have higher susceptibility to hypoxia-driven inflammation (44) and vascular and intestinal barrier dysfunction (16, 45). Used to treat IBD, the medications methotrexate and sulfasalazine exert part of their anti-inflammatory effects via CD73-mediated adenosine generation (46, 47). Medications that increase conversion of ATP and ADP to adenosine yield therapeutic benefits in ischemia and inflammatory disorders (38, 48–51).

### Extracellular Adenosine Signaling

Extracellular adenosine signals through one of four G-protein coupled seven membrane spanning cell surface receptors:A1AR, A2AAR, A2BAR, and A3AR (1, 52–54). Adenosine receptor subtypes are differentially expressed in each target cell. A2AAR is greatly expressed on immune cells like neutrophils (55) and lymphocytes (56) while A2BAR is strongly expressed on vascular endothelial cells (57). Adenosine receptor knockout mice are viable and there are no known human diseases associated with adenosine receptor mutations and defects. However, adenosine receptor functions have been elucidated in many pathologic states. For example, adenosine's chronotropic effects via A1AR is essential in the treatment of supraventricular tachycardia (58). A2AAR serves anti-inflammatory functions in neutrophils, diminishing inflammatory cell activation at various sites (55, 59). A2AAR antagonists exert benefits in Parkinson's disease (60). A2BAR contribute to tissue adaptation in response to inflammation, ischemia, and hypoxia (51, 61–63). A3AR functions in aqueous humor production in the eye (64), and agonism of A3AR has proven effective in the treatment of dry eye (65).

### Termination of Adenosine Signaling

Extracellular adenosine can be transported into the cell via concentrative or equilibrative nucleoside transporters known as CNTs and ENTs. Diffusion-limited, these channels allow adenosine to diffuse freely across the cellular membrane, following its concentration gradient (24). Adenosine movement into intracellular space diminishes adenosine signaling (66). Adenosine signaling can also be terminated by deamination of extracellular adenosine to inosine by cell surface CD26-conjugated adenosine deaminase (ADA) (67, 68) or via phosphorylation back into AMP via adenosine kinase (23). Genetic deficiency of ENTs is not lethal. ENT-deficient mice exhibit elevated adenosine levels that provide protection during disease states like organ ischemia (69). Pharmacologic blockade of ENT with dipyridamole, resulting in accumulation of extracellular adenosine causing coronary artery vasodilation, is employed in stress echocardiography to identify coronary atherosclerotic lesions (70). ENT antagonism is also used to inhibit platelet aggregation and prevent recurrence of stroke (71) and to preserve the patency of hemodialysis grafts (72). ADA-deficient mice exhibit elevated extracellular adenosine levels, which result in severe pulmonary inflammation and fibrosis (73). In human, a defect in the ADA gene causes severe combined immunodeficiency (SCID) resulting from metabolites of adenosine exerting cytotoxic effects on lymphocytes. ADA-associated SCID has been successfully treated with ADA gene therapy (74). The anti-inflammatory effects of cyclosporine may be partially due to inhibition of adenosine kinase, resulting in elevated adenosine levels (75).

## Purinergic Signaling in Acute Pulmonary Inflammation

**Figure 3:**
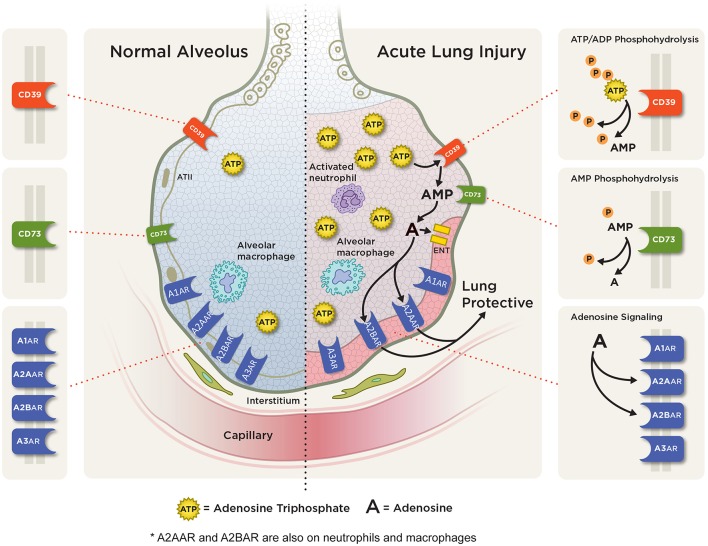
Adenosine signaling in acute pulmonary inflammation. ATP and ADP (not shown) are released into the extracellular space from various cells during pulmonary injury. Extracellular ATP is rapidly metabolized to AMP via CD39 and then to adenosine via CD73. In acute lung injury/ARDS, adenosine can signal through one of four adenosine receptors: the A1AR, A2AAR, A2BAR, and A3AR with A2AAR and A2BAR receptor expression being induced during hypoxic and inflammatory conditions on several cell types including alveolar epithelial cells, neutrophil, and macrophages. Studies have shown an anti-inflammatory, tissue protective role with attenuation of pulmonary inflammation and edema with A2AAR and A2BAR adenosine signaling.

Extracellular nucleotides and nucleosides (particularly ATP and adenosine) along with the ectonucleotidases CD73 and CD39 (responsible for the conversion of ATP to adenosine) and nucleoside transporters (ENTs) have been demonstrated to play essential roles in the pathogenesis of acute pulmonary inflammation (Figure 3). We will examine their roles below.

### Nucleotides in Acute Pulmonary Inflammation

Extracellular ATP is elevated in acute pulmonary inflammation and has been shown to play an essential role in disease pathogenesis, though that role is still controversial. Using a mouse model of LPS-induced acute lung inflammation, Kolosova et al. demonstrated the role of ATP in reducing pulmonary inflammation and enhancing endothelial cell barrier function (76). Dagvador et al. reported that LPS activation of the P2X7 receptor on alveolar macrophages (AM) promoted depletion of ATP, leading to necrosis. These necrotic AMs then release pro-interleukin 1α (IL-1α), which enhance vascular permeability via activation of endothelial cells and loss of vascular endothelial cadherin. Deficiency of P2X7R attenuated AM necrosis and pro-IL-1α secretion (77). Using adoptive transfer techniques in a murine hyperoxia model of acute lung injury, Nowak-Machen et al. reported tissue protective effects of ATP-mediated P2X7 receptor activation. They demonstrated an essential role for pulmonary invariant natural killer T (iNKT) cells in the pathogenesis of hyperoxia-induced acute lung injury. They showed that the ectonucleotidase CD39 is highly expressed in iNKT cells and regulates activation of these cells in the model. Absence of CD39 and iNKT were protective against hyperoxic lung injury. They revealed ATP-induced purinergic signaling mediates iNKT cell death and specific blockade of P2X7 receptor signaling induces hyperoxic pulmonary inflammation (78). While some studies show an anti-inflammatory role for ATP, Matsuyama et al. reported an opposing role. They observed that ATP concentration is increased in bronchial alveolar lavage fluid (BALF) from lungs with pulmonary inflammation caused by high tidal volume mechanical ventilation. ATP induces pulmonary inflammation in these lungs via the P2Y receptor and specific antagonism of P2 receptor partially attenuated the inflammatory response, suggesting a partial role for the ATP-P2Y receptor system in ventilator-induced pulmonary inflammation (79). Moreover, P2Y6 receptor has been shown to be induced in endothelial cells upon LPS exposure and its induction results in increased vascular inflammation (34). To summarize, the function of ATP and its receptors are diverse in acute pulmonary inflammation dependent on their roles in different cell types and disease conditions.

In addition to its critical role in inflammation, ATP and its receptors are also crucial on alveolar surfactant maintenance and secretion as well as the regulation of microbiome during acute pulmonary diseases. Pulmonary surfactant plays a central role in acute and chronic lung disease (80). ATP and ATP receptors have been shown to be important in surfactant production and secretion (81, 82). Mechanical stretch of alveolar type I (AT I) cells resulted in the release of high levels of extracellular ATP, which desensitized the ATP receptors on alveolar type II (AT II) cells leading to impairment in surfactant production. The reduced surfactant, in turn, collapsed the alveoli and exaggerated the increase in extracellular ATP. Subsequently, high extracellular ATP levels (>300 μM) exacerbated pulmonary edema and acute lung injury (83). Moreover, the paracrine regulation of surfactant production between AT I and AT II cells has been indicated as AT II cells secrete surfactant lipid in response to tonic stretch only in the presence of AT I cells. This study further demonstrated that ATP releases by the AT I cells were crucial in this response as treatment of apyrase and adenosine deaminase abolished this phenomenon (84). Further study has indicated that activation of the P2X7 receptor resulted in the release of ATP from AT I cells which subsequently activated the P2Y2 receptor in AT II cells. Hyperventilation elevated surfactant secretion however P2X7^−/−^ mice lost the response, suggesting the importance of P2X7 receptor in surfactant maintenance (85). Lung microbiome has been underneath the spotlight recently to be associated with the pathogenesis of ARDS (86) and ATP regulates lung microbiome via several different mechanisms. For instance, Marks et al. have shown that ATP induces the escape of *Streptococcus pneumoniae* from biofilms during both *in vitro* and *in vivo* colonization, suggesting a crucial role of interkingdom signaling to regulate microbe dispersion (87). ATP has also been shown to confer iron-chelating ability which resulted in the growth inhibition of various bacteria such as Staphylococcus, Pseudomonas, and mycobacteria (88). In conclusion, ATP and its receptors have a multi-faceted role in acute pulmonary diseases and infections by regulating surfactant homeostasis and lung microbiome.

### Ectonucleotidase in Acute Pulmonary Inflammation

CD39 (ecto-apyrase) and CD73 (ecto-5′-nucleotidase) act to shift the balance from ATP signaling to adenosine signaling via enzymatic conversion of ATP and ADP to AMP, then to adenosine. In an intratracheal LPS murine model of acute pulmonary inflammation, genetic deficiency of CD73 led to greater mortality and impairment in resolution. The study revealed levels of CD73 are highest in regulatory T cells (Tregs) during lung injury and presence of CD73 is necessary for proper immunosuppressive functions of Tregs. Adoptive transfer studies suggest CD73-mediated generation of adenosine in Tregs is essential in resolution of acute pulmonary inflammation (20). Reutershan et al. (48) demonstrated an essential role of CD39 and CD73 in neutrophil transmigration in response to LPS injury in the lung. Levels of pulmonary CD39 and CD73 transcripts were increased after LPS. Genetic deficiency of CD39 or CD73 resulted in enhanced pulmonary PMN accumulation, along with changes in barrier permeability, in response to LPS injury. Inhibition of phosphohydrolysis of extracellular nucleotides using Sodium polyoxotungstate (POM-1) resulted in enhanced pulmonary PMN accumulation in wild-type but not knockout mice. Replacement of apyrase or nucleotidase in genetically deficient mice decreased neutrophil accumulation and pulmonary edema after LPS administration. In a ventilator-induced model of acute pulmonary inflammation, pulmonary adenosine levels were increased with mechanical ventilation. CD39 and CD73 are responsible for the generation of extracellular adenosine and both were increased with mechanical ventilation. CD39 deficient mice that were mechanically ventilated showed enhanced pulmonary edema and inflammation. Genetic deficiency or pharmacologic inhibition of CD73 worsened ventilator-induced lung inflammation. Administration of soluble CD39 or CD73 to deficient mice rescued the phenotypes. Moreover, the role of CD73 mediated production of adenosine on endothelial barrier function has been harnessed in the clinic. Bellingan et al. demonstrated IFN-beta-1a up-regulated CD73 production in *ex vivo* studies of human lung tissues. They then conducted a phase I/II, open-label study to test the safety, tolerability, and efficacy of intravenous human recombinant IFN-beta-1a (FP-1201) in ARDS patients. After a dose-escalation phase to determine optimal tolerated dose, ARDS patients were treated with FP-1201 for 6 days and followed for the primary endpoint of 28-day mortality. The trial revealed human IFN-beta-1a was well tolerated and treatment led to an 81% reduction in odds of 28-day mortality (89). These findings are now being substantiated in a larger phase III double-blind randomized controlled clinical trial examining treatment with human IFN-beta-1a in moderate to severe ARDS (90). These findings revealed a protective role for CD39 and CD73 in the lung during acute pulmonary inflammation.

### Adenosine in Acute Pulmonary Inflammation

In acute pulmonary inflammation, elevated extracellular adenosine promotes tissue-protective responses to hypoxia, including dampening inflammation, augmenting tissue tolerance of ischemia, and re-establishing normal oxygenation. Studies have shown beneficial effects of adenosine in the acute phase of lung injury (91–93). In a murine model of intratracheal LPS-induced pulmonary inflammation, treatment with adenosine or NECA (5'-N-ethylcarboxamidoadenosine—non-selective AR agonist), resulted in a decrease in inflammatory response and improvement of barrier function, as seen by decreased cell counts, Evans blue dye albumin (EBDA) extravasation, protein and cytokine levels, and reduced neutrophil infiltration. Adenosine prevents LPS-induced protein degradation of A2AAR and A3AR (94). Administration of peroxisome proliferator-activated receptors γ (PPARγ) and A2AAR agonists resulted in a synergistic effect with regards to attenuating pulmonary inflammation and edema, improving gas exchange and pulmonary function in a murine model of LPS-induced pulmonary inflammation. PPARγ and A2AAR were shown to regulate each other's expression. PPARγ binds a DR10 response element (−218 to −197) of A2AAR gene promoter leading to upregulation of receptor expression. A2AAR signals via protein kinase A (PKA)-cAMP response element binding protein (CREB), promoting CREB binding to cAMP-responsive element (CRE)-like site within PPARγ gene promoter, thus upregulating PPARγ expression (95). Similarly, in an LPS-induced murine model of pulmonary inflammation, induction of A2BARwas seen in response to LPS. This induction occurred via mRNA stability. Genetic deficiency or pharmacologic blockade of A2BAR led to an exacerbation of pulmonary inflammation and tissue injury. Studies using A2BARbone marrow chimeric mice supported a lung protective role for A2BAR signaling. Administration of BAY 60-6583, a specific agonist of A2BAR, resulted in a reduction in pulmonary inflammation and pulmonary edema (91). In a murine model of intra-tracheal LPS-induced pulmonary inflammation, CD73 expression was significantly elevated in lymphoid cells while CD39 was highly abundant on myeloid cells. CD39, CD73, and A2AAR were significantly elevated in T helper cells. Specifically, CD4 T cells of injured lungs generate adenosine from ATP at an accelerated rate. Since pulmonary T cells are the dominant cell type present in the later phase of acute pulmonary inflammation, the increase in adenosine along with upregulation of A2BAR likely govern the repair process after acute injury (8).

Prior studies have supported a lung protective role for adenosine signaling through A2BAR. In a two-hit model of acute pulmonary inflammation that involves intra-tracheal LPS instillation combined with detrimental mechanical ventilation, Hoegl et al. demonstrated that tissue-specific A2BAR signaling, namely in alveolar epithelial cells, mediates this protective role. Specific deletion of A2BAR in alveolar epithelial cells, in comparison to myeloid and endothelial deletions, significantly attenuated pulmonary inflammation and edema (96). Adenosine levels are increased in mechanically ventilated lungs. Using cyclic mechanical stretch to mimic ventilator-induced inflammation, Eckle et al. demonstrated a significant selective induction of A2BAR in pulmonary epithelial cells, an increase mediated by hypoxia-inducible factor 1 (HIF-1). Genetic or pharmacologic inhibition of HIF1α prevents A2BAR induction, supporting a role for HIF in transcriptional control of adenosine signaling in ALI (97). Using a murine model of oleic acid (OA)-induced acute pulmonary inflammation, Xu et al. demonstrated that activation of A2BAR, using a selective agonist (BAY60-6583), resulted in decreased AEC apoptosis, an effect that was abolished with the administration of a selective A2BAR antagonist. Studies of hydrogen peroxide (H_2_O_2_)-induced AEC injury revealed lower AEC apoptosis rates with A2BAR activation, with the mechanism of action via suppression of p38 and ERK1/2-mediated mitochondrial apoptosis pathway (98). Furthermore, a study by Koscso et al. demonstrated that the activation of A2BAR conferred lung protection in trauma-hemorrhagic shock-induced lung injury (99). Indeed, treatment of A2BARagonist BAY 60-6583 resulted in attenuated lung injury marked by reduced lung permeability and creatine kinase levels in the plasma. In summary, adenosine facilitates lung protection during murine models of acute pulmonary inflammation through A2BAR.

Adenosine signaling is also crucial in endothelial barrier function during acute lung inflammation. Lu et al. showed that adenosine confers a dose-dependent improvement in endothelial barrier function, an effect that was partially abolished with antagonism by either an adenosine transporter inhibitor (NBTI) or a combination of A2AAR and A2BAR antagonists (DPMX and MRS1754) (100). RNA silencing of both A2AAR and A2BAR also resulted in a partial reversal of the effect on barrier function, while NECA administration, which activates both A2AAR and A2BAR, improved barrier function. Treatment with both adenosine transporter inhibitor and A2AAR/A2BAR antagonists completely abolished adenosine's effect on barrier function, suggesting both are required for adenosine to exert a maximum effect on barrier function. Adenosine enhances barrier function via increased Rac1 GTPase activity. Treatment with Pentostatin, to inhibit adenosine deaminase and increase adenosine levels, led to the enhancement of barrier function, via increased activity of Rac1 GTPase leading to elevated focal adhesion complexes and adherens junctions. Using a non-inflammatory alpha-naphthylthiourea-induced model of acute lung inflammation, the authors showed treatment with Pentostatin increased pulmonary adenosine level leading to both a reduction in edema development and partial reversal of edema. Furthermore, the important role of A2BAR on macrophages has been identified during vascular stress. Indeed, macrophages upregulated A2BAR in mice with arterial injury and the expression of A2BAR dampens the TNF-α production (101). These studies indicate the important role of adenosine signaling in vascular injury and inflammation during acute pulmonary inflammation.

Besides A2AAR and A2BAR, the activation of other adenosine receptors has been shown to modulate pulmonary inflammation following ischemia-reperfusion (IR) injury as well as influenza infection. For instance, the A3AR is present in pulmonary tissue and inflammatory cells. Activation of A3AR with a selective agonist diminishes pulmonary inflammation and edema, cytokine levels and neutrophil chemotaxis and activation. This effect is not seen in *A3A*R^−/−^ mice (102). While the receptors A2AAR, A2BAR, and A3AR mediate lung protective effects in acute injury, signaling through A1AR is detrimental. Adenosine production is increased in acute lung inflammation following influenza infection (103). Adenosine activation of the A1AR promotes pulmonary recruitment of innate immune cells and progression of pulmonary injury. Antagonism of A1AR attenuates pulmonary injury in influenza-infected mice. Follow up study pinpointed the important role of ATP catabolism by tissue-nonspecific alkaline phosphatase (TNAP) on the generation of adenosine during influenza infection. TNAP increases following influenza infection and treatment of TNAP inhibitor significantly attenuated pulmonary injury in influenza-induced ARDS (104).

### Adenosine Signaling Termination in Acute Pulmonary Inflammation

Adenosine signaling termination occurs via regulation of adenosine levels by equilibrative nucleoside transporters (ENTs), adenosine kinase (AK), and adenosine deaminase (ADA). Using high-pressure mechanical ventilation to induce lung inflammation, Eckle et al. reported ENT1 and ENT2 repression in the injured lung. Treatment with the ENT inhibitor dipyridamole significantly prolonged survival. Studies in genetically deficient mice demonstrated a phenotype in ENT2^−/−^ mice that includes elevated adenosine associated with diminished pulmonary edema and improved gas exchange. ENT-dependent lung protection was mediated by A2BAR activation in alveolar epithelial cells (105). Repression of ENT1 and ENT2 (with NF-κB being a key regulator), is associated with increased extracellular adenosine due to decreased uptake. Pharmacologic blockade of ENT1 and ENT2 in LPS-induced murine model of lung inflammation attenuated pulmonary inflammation and enhanced barrier function (106).

Inhibition of ENTs and adenosine kinase elevates extracellular adenosine levels and increase adenosine signaling. In a murine model of LPS-induced lung injury, adenosine kinase was repressed by pro-inflammatory cytokines and nuclear factor kappa-light-chain-enhancer of activated B cells (NF-κB) influenced regulation of adenosine kinase promoter. Mice with repressed adenosine kinase (AK^±^), when subjected to LPS exposure, exhibited attenuated pulmonary inflammation and decreased vascular permeability as evidenced by a reduction in the transmigration of neutrophils into alveolar space and decreased total protein, myeloperoxidase, and cytokine levels in BALF. Additionally, pharmacologic inhibition of AK, with a subsequent increase in extracellular adenosine levels, produced similar results (107). Adenosine deaminase inhibition also increases extracellular adenosine. Ehrentraut et al. reported the administration of peg-ADA to reduce extracellular adenosine levels in an LPS-induced mouse model of lung injury compromised resolution of pulmonary inflammation (20). Thus, ENTs, AK, and ADA modulate adenosine levels and terminate adenosine signaling, resulting in exaggerated pulmonary inflammation.

## Purinergic Signaling in Chronic Pulmonary Inflammation

**Figure 4:**
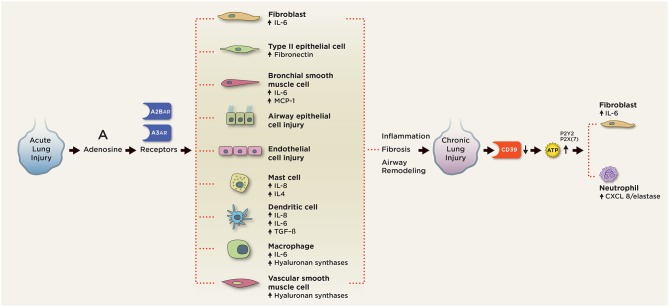
Purinergic signaling in chronic pulmonary inflammation. In chronic lung injury, elevated extracellular ATP activates P2 receptors on neutrophils to release CXCL3/elastase and fibroblasts to release IL-6. Elevated extracellular adenosine signals through the A2BAR and A3AR on various pulmonary cell types to induce aberrant cell differentiation and production of pro-inflammatory, pro-fibrotic mediators, including IL-4, IL-6, IL-8, fibronectin, and TGF-beta. A2BAR engagement on mast cells induces production of IL-4, IL-8, IL-13, and VEGF. A2BAR activation promotes fibroblast and myofibroblast proliferation and differentiation. Signaling through A2BAR led to the production of IL-6 and osteopontin from macrophages, MCP-1, and IL-6 from bronchial smooth muscle cells, IL-6 release from fibroblasts, fibronectin expression in type 2 airway epithelial cells (AECs), and hyaluronan synthetases from macrophages and vascular smooth muscle cells. A2BAR signaling is responsible for the maintenance of vascular barrier integrity in endothelial cells.

ATP, adenosine, CD73, and CD39 have also been implicated in the pathogenesis of chronic lung inflammation (Figure 4). We will examine their roles below.

### Nucleotides in Chronic Pulmonary Inflammation

Extracellular ATP is elevated in chronic lung diseases, like asthma, COPD and IPF, and contributes to disease development and progression. Allergen challenge in both asthmatic human patients and experimental mouse asthma models causes acute accumulation of ATP in the airways. Eosinophilic inflammation, Th2 cytokine production, and airway reactivity were abrogated when ATP was depleted using apyrase or the P2 receptor was blocked in mice (35). Smokers with COPD were found to have a strong and persistent up-regulation of ATP in airway space through BAL fluid analysis (108). When compared to healthy subjects, smokers, and COPD patients also have increased sensitivity to ATP, experiencing more dyspnea, cough and throat irritation in response to inhaled ATP and AMP (109). In cigarette smoke mouse models of emphysema, ATP was increased in the BAL of exposed mice, which resulted in the activation of neutrophils and release of CXCL8 and elastase (110).

Extracellular ATP levels were elevated in the lungs of patients with IPF in mice with bleomycin-induced pulmonary fibrosis, in association with upregulation of P2Y2 receptor expression (111, 112). Pulmonary epithelial cells secrete ATP in response to bleomycin injury, a process partially dependent on P2X(7) receptor and pannexin-1 (112). Effects of extracellular ATP on inflammation and fibrosis are mediated through P2X(7) receptor/pannexin-1 as P2X(7) receptor deficiency resulted in attenuation of pulmonary inflammation and fibrosis after bleomycin exposure (112). Pulmonary fibrosis was attenuated in P2Y2-deficient mice via a reduction in neutrophil recruitment, migration, and proliferation of fibroblasts and interleukin 6 (IL-6) production (111). Pharmacologic reduction of ATP levels (using apyrase, an ATP-degrading enzyme) significantly attenuated pulmonary inflammation, decreased various mediators, including interleukin 1 beta (IL-1-β), and tissue inhibitor of metalloproteinase 1 (TIMP-1) production. Conversely, the artificial elevation of ATP levels (via an ATP derivative, ATP- γS) worsened pulmonary inflammation in response to bleomycin (112). Thus, increased ATP is detrimental in chronic lung diseases/inflammation.

### Ectonucleotidase in Chronic Pulmonary Inflammation

CD39 and CD73 serve to decrease extracellular ATP levels but increase extracellular adenosine levels, thus shifting the balance from ATP signaling to adenosine signaling. There are conflicting reports of the effects of CD39 and CD73 in chronic lung diseases. CD39 converts ATP and ADP to AMP. Correlating with increased ATP levels in the airways of COPD smokers, there was a significant decrease in CD39 gene and protein expression and ATPase activity in lung tissue acquired from COPD patients when compared with non-obstructive smokers and never-smokers (113). CD39 is decreased on T-cells in acute exacerbation of COPD (AECOPD) patients when compared to stable COPD. Higher CD39 was correlated with increased plasma soluble Tumor necrosis factor alpha (TNF-α) receptor, resulting in impaired T-cell responses (114). CD39 deficiency in mice leads to enhanced emphysema in cigarette smoke mouse models (115). CD73 metabolizes AMP to adenosine. COPD and IPF patients exhibit increased CD73 activity (116) along with elevated adenosine levels (117, 118). CD39 and CD73 levels correlated with pulmonary hypertension severity in explanted lungs from patients with IPF (119). CD73 potentiates radiation-induced lung fibrosis (120).

### Adenosine in Chronic Pulmonary Inflammation

As opposed to its beneficial anti-inflammatory role in acute disease states, elevated adenosine levels in the chronic setting promotes detrimental tissue injury and fibrosis. In humans and mouse models, adenosine has been implicated in the development and progression of chronic lung disorders, including asthma, COPD, and IPF (11). Elevated adenosine levels were initially reported in bronchoalveolar lavage fluid and then later confirmed in exhaled breath condensate from asthmatics (121, 122). Levels of adenosine are increased after allergen exposure in asthmatics (123). Adenosine was shown to induce airway hyper-responsiveness, causing bronchoconstriction, in patients with asthma (124) and COPD (125). Adenosine levels are also elevated in BALF and exhaled breath condensate of patients with COPD (116, 126) and are negatively correlated with pulmonary function.

Extracellular adenosine levels have been difficult to measure in patients with interstitial lung disease and IPF, likely due to its lability. However, adenosine is elevated in mouse models of interstitial lung disease and pulmonary fibrosis (127–129). In the bleomycin-induced mouse models of pulmonary fibrosis, extracellular adenosine levels are elevated in association with increasing pulmonary fibrosis and levels decrease with the resolution of disease. Administration of dipyridamole, a nucleoside transporter inhibitor, to potentiate elevated adenosine levels resulted in the failure of pulmonary fibrosis to resolve and in fact exacerbates fibrosis (130). Elevated adenosine levels were seen in association with elevated IL-6 and interleukin 17 (IL-17) levels, which have been shown to be crucial inflammatory, pro-fibrotic mediators in pulmonary fibrosis (130–132).

Studies of chronic lung diseases using animal models suggested antagonism of A1AR, A3AR, A2BAR, and perhaps even A2AAR, may be beneficial in the treatment of asthma and COPD (133). Recent publications have reported, though, that it is the activation of the A2B receptor by adenosine that mediates production of pro-inflammatory, pro-fibrotic molecules, like interleukin-6, leading to chronic lung diseases, particularly pulmonary fibrosis (11, 118, 132, 134, 135). The A2BAR is elevated in COPD and IPF (118). A2BAR is increased in remodeled airway epithelial cells in a rapidly progressing variant of IPF (136). Genetic removal of A2BAR in a mouse model of asthma diminished allergen-induced chronic lung inflammation and airway remodeling. In response to allergen provocation, A2BAR^−/−^ mice experienced a reduction in eosinophilic recruitment and infiltration and attenuation of interleukin 4 (IL-4) and TGF-beta release, which translated to reduced airway smooth muscle and goblet cell hypertrophy and hyperplasia (137). Genetic deficiency of A2BAR in myeloid cells attenuated pulmonary fibrosis, improved respiratory function and prevented the development of pulmonary hypertension in response to bleomycin exposure. Specifically, there was a reduction in alternatively activated macrophages, IL-6, and hyaluronan, all of which have been implicated in pulmonary fibrosis and pulmonary hypertension. The study suggests that activation of the A2BAR on alternatively activated macrophages is essential in the development of pulmonary fibrosis and pulmonary hypertension (138). In addition, A2BAR activation on pulmonary artery smooth muscle cells (PASMCs) results in increased IL-6 and hyaluronan, and deletion of A2BAR in these cells leads to reduced severity of pulmonary hypertension (139). Moreover, hypoxia is present in pulmonary fibrosis and its role in modulating alveolar macrophage phenotype was examined by Philip et al. Using the mouse bleomycin-induced model of pulmonary fibrosis and IPF lung samples, they determined that inhibition of HIF1α resulted in a reduction in pulmonary fibrosis, along with the diminished expression of A2BAR in alternatively activated macrophages (AAMs). HIF1α inhibition in combination with A2BAR deletion prohibited differentiation of AAM and subsequent IL-6 production and release from AAMs (140). Thus, hypoxia regulates A2BAR expression on AAMs, differentiation of macrophages into AAMs and production of pro-fibrotic mediators like IL-6. Zhong et al. went on to show there is a synergism between hypoxia and A2BAR in that hypoxia upregulates A2BAR and amplifies adenosine's effect on IL-6 release and differentiation of fibroblasts to myofibroblasts, which are essential in development and progression of lung fibrosis (141). In summary, chronic adenosine signaling promotes pulmonary inflammation and fibrosis.

### Adenosine Signaling Termination in Chronic Pulmonary Inflammation

Adenosine deaminase converts adenosine to inosine. COPD and IPF patients exhibit the reduced activity of ADA along with elevated adenosine levels (117, 118). ADA expression correlated inversely with pulmonary hypertension severity in explanted lungs from patients with IPF (119). Genetic deficiency of ADA in mice led to the spontaneous and progressive development of pulmonary fibrosis due to an accumulation of extracellular adenosine. Examination of the pulmonary phenotype seen in ADA-deficient mice suggests that elevated adenosine activates signaling pathways that lead to and worsen chronic lung diseases (142). Using ADA-deficient mice, Sun et al. reported that selective pharmacologic antagonism of the A2BAR led to attenuation of pulmonary inflammation, emphysema, and fibrosis. A2BAR blockade decreased levels of proinflammatory, profibrotic mediators. The same findings were seen with selective antagonism of A2BAR in the bleomycin-induced mouse model of pulmonary fibrosis (143). Thus, the termination of adenosine signaling via ADA activity is protective in chronic lung inflammation.

## Discussion and Perspectives

Purinergic signaling serves an essential regulatory role in a number of inflammatory conditions. Many of the studies presented in this review support targeting purinergic signaling as a therapeutic approach to the treatment of acute lung diseases like ARDS and chronic lung diseases like asthma, COPD, and IPF. However, certain gaps in knowledge remain and need to be addressed. For example, the role of ATP in acute pulmonary injury and inflammation is controversial and needs to be addressed with further studies. A role for ENTs has been reported in acute injury but not in chronic lung diseases. Further research into this area may prove beneficial as ENTs regulate the metabolism of adenosine and hence affect adenosine signaling in the lungs. A better understanding of when and where purinergic signaling serves protective roles and when and where it is detrimental, as well as translation of findings from mice to humans, is vital to developing therapies to combat acute and chronic pulmonary diseases in patients. In the meanwhile, targeting purinergic/adenosinergic pathway is feasible (53, 144) and could be of great therapeutic potential to prevent and treat acute and chronic lung inflammation.

## Author Contributions

T-TL drafted the manuscript. NB assisted with the literature search and drafted the figures. MH drafted the figures. XL assisted with the literature search. HE revised the manuscript and provided critical advice on the structure and content of the manuscript. XY revised and finalized the manuscript.

### Conflict of Interest Statement

The authors declare that the research was conducted in the absence of any commercial or financial relationships that could be construed as a potential conflict of interest.
